# Supplementary material for: Heat adaptation of phage T7 under an extended genetic code
Source: Virus Evol. 2021 Dec 1;7(2):veab100. doi: 10.1093/ve/veab100 (PMC8923235; doi:10.1093/ve/veab100)
Supplement: veab100_Supp [file veab100_supp.zip › Sequencing_Analysis.zip!/Sequencing_Analysis/S3/evidence/JC_SIDE_1_53.html]

BRESEQ :: Evidence


| New junction evidence | | | | | | | | | | | |
| --- | --- | --- | --- | --- | --- | --- | --- | --- | --- | --- | --- |
|  | | seq id | position | reads (cov) | reads (cov) | score | skew | freq | annotation | gene | product |
| \* | ? | NC\_001604.1 | = 16604 | 551 (0.320) | 655 (0.390) | 233/590 | 1.5 | 51.6% | intergenic (–/–) | *–/–* | –/– |
|  | ? | NC\_001604.1 | 17028 = | 688 (0.410) |  |  |  |  | intergenic (–/–) | *–/–* | –/– |

Only 100 of 1275 total aligned reads displayed.  

`GGGTACATGATGAAATCCAAGTAGGCTGCCGTACCGAAGAGATTGCTCAGGTGGTCATTGAGACCGCACAAGAAGCGATGCGCTGGGTTGGAGACCACTGGAACTTCCGGTGTCTTCTGGATACCGAAGGTAAGATGGGTCCTAATTGGGCGATTTGCCACTGATACAGGAGGCTACTCATGAACGAAAGACACTTAACAGGTGCTGCTTCTGAAATGCTAGTAGCCTACAAATTTACCAAAGCTGGGTACACTGTCTATTACCCTATGCTGACTCAGAGTAAAGAGGACTTGGTTGTATGTAAGGATGGTAAATTTAGTAAGGTTCAGGTTAAAACAGCCACAACGGTTCAAACCAACACAGGAGATGCCAAGCAGGTTAGGCTAGGTGGATGCGGTAGGTCCGAATATAAGGATGGAGACTTTGACATTCTTGCGGTTGTGGTTGACGAAGATGTGCTTATTTTCACATGGGACGAAGTAAAAGGTAAGACATCCATGTGTGTCGGCAAGAGAAACAAAGGCATAAAACTATAGGAGAAATTATTATGGCTATGACAAAGAAATTTAAAGTGTCCTTCGACGTTACCGCAAAGATGTCG  >  NC_001604.1/16304‑16904`  

                                                                                                                                                                                                                                                                                                            `|`                                                                                                                                                                                                                                                                                                              
`gggTACATGATGAAATCCAAGTAGGCTGCCGTACCGAAGAGATTGCTCAGGTGGTCATTGAGACCGCACAAGAAGCGATGCGCTGGGTTGGAGACCACTGGAACTTCCGGTGTCTTCTGGATACCGAAGGTAAGATGGGTCCTAATTGGGCGATTTGCCACTGATACAGGAGGCTACTCATGAACGAAAGACACTTAACAGGTGCTGCTTCTGAAATGCTAGTAGCCTACAAATTTACCAAAGCTGGGTACACTGTCTATTACCCTATGCTGACTCAGAGTAAAGAGGACTTGGTTGTATG                                                                                                                                                                                                                                                                                                              >  1:269760‑M1/1‑301 (MQ=255)`  
`gggTACATGATGAAATCCAAGTAGGCTGCCGTACCGAAGAGATTGCTCAGGTGGTCATTGAGACCGCACAAGAAGCGATGCGCT‑GGTTGGAGACCACTGGAACTTCCGGTGTCTTCTGGATACCGAAGGTAAGATGGGTCCTAATTGGGCGATTTGCCACTGATACAGGAGGCTACTCATGAACGAAAGACACTTAACAGGTGCTGCTTCTGAAATGCTAGTAGCCTACAAATTTACCAAAGCTGGGTACACTGTCTATTACCCTATGCTGACTCAGAGTAAAGAGGACTTGGTtgtatgt                                                                                                                                                                                                                                                                                                             <  1:117270/301‑1 (MQ=255)`  
     `acagatgaAATCCAAGAAGGCTGCCGTACCGAAGAGATTGCTCAGGTGGTCATTGAGACCGCACAAGAAGCGATACGCTGGGTTGGAGACCACTGGAACTTCCGGTGTCTTCTGGATACCGAAGGTAAGATGGGTCCTAATTGGGCGATTTGCCACTGATACAGGAGGCTACTCATGAACGAAAGACACTTAACAGGTGCTGCTTCTGAAATGCTAGTAGCCTACAAATTTACCAAAGCTGGGTACACTGTCTATTACCCTATGCTGACTCAGAGTAAAGAGGACTTGGTTGTATGTAAgg                                                                                                                                                                                                                                                                                                         <  1:306393/298‑1 (MQ=255)`  
             `agtccAAGTGGGCTGCCGTACCGAAGAGATTGGACAGGTGGGAGTTGGGACCGCACAAGAAGCGATGCGCTGGGTTGGAGACCACTGGAACTTCCGGTGGCTTCTGGATAACGAAAGTAAGATGTGTCCTAATTGGGCGATTTGCCACTGATACAGGAGGCTACTCATGAACGAAAGACACTTAACAGGTGCTGCTTCTGAAATGCTAGTAGCCTACAAATTTACCAAAGCTGGGTACACTGTCTATGACCCTATGCTGACTCAGAGTAAAGAGGACTTGGTTGTATGgaaggattgatga                                                                                                                                                                                                                                                                                                 <  1:150010‑M1/299‑14 (MQ=255)`  
             `aaTCCAAGTAGGCTGCCGTACCGAAGAGATTGCTCAGGTGGTCATTGAGACCGCACAAGAAGCGATGCGCTGGGTTGGAGACCACTGGAACTTCCGGTGTCTTCTGGATACCGAAGGTAAGATGGGTCCTAATTGGGCGATTTGCCACTGATACAGGAGGCTACTCATGAACGAAAGACACTTAACAGGTGCTGCTTCTGAAATGCTAGTAGCCTACAAATTTACCAAAGCTGGGTACACTGTCTATTACCCTATGCTGACTCAGAGTAAAGAGGACTTGGTTGTATGTAAGGATGGTaaa                                                                                                                                                                                                                                                                                                 <  1:100589/301‑1 (MQ=255)`  
              `aTCCAAGTAGGCTGCCGTACCGAAGAGATTGCTCAGGTGGTCATTGAGACCGCACAAGAAGCGATGCGCTGGGTTGGAGACCACTGGAACTTCCGGTGTCTTCTGGATACCGAAGGTAAGATGGGTCCTAATTGGGCGATTTGCCACTGATACAGGAGGCTACTCATGAACGAAAGACACTTAACAGGTGCTGCTTCTGAAATGCTAGTAGCCTACAAATTTACCAAAGCTGGGTACACTGTCTATTACCCTATGCTGACTCAGAGTAAAGAGGACTTGGTTGTATGgaaggattgatgac                                                                                                                                                                                                                                                                                                >  1:263521‑M1/1‑287 (MQ=255)`  
                `ccAAGTAGGCTGCCGTACCGAAGAGATTGCTCAGGTGGTCATTGAGACCGCACAAGAAGCGATGCGCTGGGTTGGAGACCACTGGAACTTCCGGTGTCTTCTGGATACCGAAGGTAAGATGGGTCCTAATTGGGCGATTTGCCACTGATACAGGAGGCTACTCATGAACGAAAGACACTTAACAGGTGCTGCTTCTGAAATGCTAGTAGCCTACAAATTTACCAAAGCTGGGTACACTGTCTATTACCCTATGCTGACTCAGAGTAAAGAGGACTTGGTTGTATGTAAGGATGGTAAAttt                                                                                                                                                                                                                                                                                              <  1:252751/301‑1 (MQ=255)`  
                     `gcggCTGCCGTACCGAAGAGATTGCTCAGGGTGTCGTTGAGGCCGCACAAGAAGCGATGCGCTGGGTTGGAGACCACTGGAACTTCCGGTGTCTTCTGGATACCGAAGGTAAGATGAGTCCTAATTGGGCGATTTGCCACTGATACAGGAGGCTACTCATGAACGAAAGACACTTAACAGGTGCTGCTTCTGAAATGCTAGTAGCCTACAAATTTACCAAAGCTGGGTACACTGTCTATTACCCTATGCTGACTCAGAATAAAGAGGACTTGGTTGTATGTAAGGATGGTAAATTTAGTaa                                                                                                                                                                                                                                                                                         <  1:6917/299‑1 (MQ=255)`  
                      `atgtgtataagagacagagattgctcaggtggtcATTGAGACCGCACAAGAAGCGATGCGTTGGGTTGGAGAACACTGGAACTTCCGGTGTCTTCTGGATACCGAAGGTAAGATGGGTCCTAATGGGGCGATTTGCCACTGATACAGGAGGCTACTCATGAACGAAAGACACTTAACAGGTGCTGTTTCTGAAATGCTAGTAGCCTACAAATTTACCAAAGCTGGGTATACTGTCTATTACCCTATGCTGACTCAGAGTAAAGAGGACTTGGTTGTATGgaaggattgatgacattcgtct                                                                                                                                                                                                                                                                                        <  1:95447‑M1/286‑23 (MQ=255)`  
                              `gTACCGAAGAGATTGCTCAGGTGGTCATTGAGACCGCACAAGAAGCGATGCGCTGGGTTGGAGACCACTGGAACTTCCGGTGTCTTCTGGATACCGAAGGTAAGATGGGTCCTAATTGGGCGATTTGCCACTGATACAGGAGGCTACTCATGAACGAAAGACACTTAACAGGTGCTGCTTCTGACATGCTAGTAACCTACAAATTTACCAAAGCTGGGTACACTGTCTATTACCCTATGCTGACTCAGAGTAAAGAGGACTTGGTTGTATGgaaggattgatgacattcgtagtacgtaca                                                                                                                                                                                                                                                                                >  1:170366‑M1/1‑271 (MQ=255)`  
                                `aCCGAAGAGATTGCTCAGGTGGTCATTGAGACCGCACAAGAAGCGATGCGCTGGGTTGGAGACCACTGGAACTTCCGGTGTCTTCTGGATACCGAAGGTAAGATGGGTCCTAATTGGGCGATTTGCCACTGATACAGGAGGCTACTCATGAACGAAAGACACTTAACAGGTGCTGCTTCTGAAATGCTAGTAGCCTACAAATTTACCAAAGCTGGGTACACTGTCTATTACCCTATGCTGACTCAGAGTAAAGAGGACTTGGTTGTATGgaaggattgatgacattcgtagtacgtacatc                                                                                                                                                                                                                                                                              <  1:127443‑M1/301‑33 (MQ=255)`  
                                        `gaTTGCTCAGGTGGTCATTGAGACCGCACAAGAAGCGATGCGCTGGGTTGGAGACCACTGGAACTTCCGGTGTCTTCTGGATACCGAAGGTAAGATGGGTCCTAATTGGGCGATTTGCCACTGATACAGGAGGCTACTCATGAACGAAAGACACTTAACAGGTGCTGCTTCTGAAATGCTAGTAGCCTACAAATTTACCAAAGCTGGGTACACTGTCTATTACCCTATGCTGACTCAGAGTAAAGAGGACTTGGTTGTATGgaaggattgatgacattcgtagtacgtacatcatttcgtg                                                                                                                                                                                                                                                                      <  1:91793‑M1/301‑41 (MQ=255)`  
                                          `ttGCTCAGGTGGTCATTGAGACCGCACAAGAAGCGATGCGCTGGGTTGGAGACCACTGGAACTTCCGGTGTCTTCTGGATACCGAAGGTAAGATGGGTCCTAATTGGGCGATTTGCCACTGATACAGGAGGCTACTCATGAACGAAAGACACTTAACAGGTGCTGCTTCTGAAATGCTAGTAGCCTACAAATTTACCAAAGCTGGGTACACTGTCTATTACCCTATGCTGACTCAGAGTAAAGAGGACTTGGTTGTATGgaaggattgatgacattcgtagtacgtacatcatttcgtgag                                                                                                                                                                                                                                                                    >  1:284018‑M1/1‑259 (MQ=255)`  
                                          `ttGCTCAGGTGGTCATTGAGA‑CGCACAAGAAGCGATGCGCTGGGTTGGAGACCACTGGAACTTCCGGTGTCTTCTGGATACCGAAGGTAAGATGGGTCCTAATTGGGCGATTTGCCACTGATACAGGAGGCTACTCATGAACGAAAGACACTTAACAGGTGCTGCTTCTGAAATGCTAGTAGCCTACAAATTTACCAAAGCTGGATACACTGTCTATTACCCTATGCTGACTCAGAGTAAAGAGGACTTGGTTATATGgaaggattgatgacattcgtagtacgtacatcatttcgtgagg                                                                                                                                                                                                                                                                   >  1:256513‑M1/1‑258 (MQ=255)`  
                                                 `ggtggtCATTGAGACCGCACAAGAAGCGATGCGCTGGGTTGGAGACAACTGGAACTTCCGGTGTCTTCTGGATACCGAAGGTAAGATGGGTCCTAATTGGGCGATTTGCCACTGATACAGGAGGCTACTCATGAACGAAAGACACTTAACAGGTGCTGCTTCTGAAATGCTAGTAGCCTACAAATTTACCAAAGCTGGGTACACTGTCTATTACCCTATGCTGACTCAGAGTAAAGAGGACTTGGTTGTATGTAAGGCTGGTAACTTTAGTAGGGTTCAGGTTAAAACAGCCACAACGGtt                                                                                                                                                                                                                                                             >  1:343240/1‑301 (MQ=255)`  
                                                    `ggtCATTGAGACCGCACAAGAAGCGATGCGCTGGGTTGGAGACCACTGGAACTTCCGGTGTCTTCTGGATACCGAAGGTAAGATGGGTCCTAATTGGGCGATTTGCCACTGATACAGGAGGCTACTCATGAACGAAAGACACTTAACAGGTGCTGCTTCTGAAATGCTAGTAGCCTACAAATTTACCAAAGCTGGGTACACTGTCTATTACCCTATGCTGACTCAGAGTAAAGAGGACTTGGTTGTATGgaaggattgatgacattcgtagtacgtacatcatttcgtgaggccattaagg                                                                                                                                                                                                                                                          >  1:197659‑M1/1‑249 (MQ=255)`  
                                                    `ggtCATTGAGACCGCACAAGAAGCGATGCGCTGGGTTGGAGACCACTGGAACTTCCGGTGTCTTCTGGATACCGAAGGTAAGATGGGTCCTAATTGGGCGATTTGCCACTGATACAGGAGGCTACTCATGAACGAAAAACACTTAACAGGTGCTGCTTCTGAAATGCTAGTAGCCTACAAATTTACCAAAGCTGGGTACACTGTCTATTACCCTATGCTGACTCAGAGTAAGGAGGACTTGGTTGTATGTAAGGGTGGTAAATTTCGTAAGGTTCAGTTTAAACCAGCCACAACGGTTcct                                                                                                                                                                                                                                                          >  1:146794/1‑299 (MQ=255)`  
                                                      `gcaTTGAGACCGCACAAGAAGCGATGCGCTGGGTTGGAGACCACTGGAACTTCCGGTGTCTTCTGGATACCGAAGGTAAGATGGGTCCTAATTGGGCGATTTGCCACTGATACAGGAGGCTACTCATGAACGAAAGACACTTAACAGGTGCTGCTTCTGAAATGCTAGTAGCCTACAAATTTACCAAAGCTGGGTACACTGTCTATTACCCTATGCTGACTCAGAGTAAAGAGGACTTGGTTGTATGgaaggattgatgacattcgtagtacgtacatcatttcgtgaggccattaaggac                                                                                                                                                                                                                                                        <  1:244727‑M1/300‑55 (MQ=255)`  
                                                       `cATTGAGACCGCACAAGAAGCGATGCGCTGGGTTGGAGACCACTGGAACTTCCGGTGTCTTCTGGATACCGAAGGTAAGATGGGTCCTAATTGGGCGATTTGCCACTGATACAGGAGGCTACTCATGAACGAAAGACACTTAACAGGTGCTGCTTCTGAAATGCTAGTAGCCTACAAATTTACCAAAGCTGGGTACACTGTCTATTACCCTATGCTGACTCAGAGTAAAGAGGACTTGGTTGTATGgaaggattgatgacattcgtagtacgtacatcatttcgtgaggccattaaggaca                                                                                                                                                                                                                                                       >  1:104932‑M1/1‑246 (MQ=255)`  
                                                          `tGAGACCGCACAAGAAGCGATGCGCTGGGTTGGAGACCACTGGAACTTCCGGTGTCTTCTGGATACCGAAGGTAAGATGGGTCCTAATT‑GGCGATTTGCCACTGATACAGGAGGCTACTCATGAACGAAAGACACTTAACAGGTGCTGCTTCTGAAATGCTAGGAGCCTACAAATTTACCAAAGCTGGGTACACTGTCTATTACCCTATGCTGACTCAGAGTAAAGAGGACTTGGTTGTATGgaaggattgatgacattcgtagtacgtacatcatttcgtgaggccattaaggacatgca                                                                                                                                                                                                                                                   <  1:144127‑M1/301‑60 (MQ=255)`  
                                                                  `cacaAGAAGCGATGCGCTGGGTTGGAGACCACTGGAACTTCCGGTGTCTTCTGGATACCGAAGGTAAGATGGTTCCTAATTGGGCGATTTGCCACTGATACAGGAGGCTACTCATGAACGAAAGACACTTAACAGGTGCTGCTTCTGAAATGCTAGTAGCCTACAAATTTACCAAAGCTGGGTACACTGTCTATTACCCTATGCTGACTCAGAGTAAAGAGGACTTGGTTGTATGgaaggattgatgacattcgtagtacgtacatcatttcgtgaggccattaaggacatgcactaagag                                                                                                                                                                                                                                            >  1:47922‑M1/1‑235 (MQ=255)`  
                                                                  `cacaAGAAGCGATGCGCTGGGTTGGAGACCACTGGAACTTCCGGTGTCTTCTGGATACCGAAGGTAAGATGGGTCCTAATTGGGCGATTTGCCACTGATACAGGAGGCTACTCATGAACGAAAGACACTTAACAGGTGCTGCTTCTGAAATCCTAGTAGCCTACAAATTTACAAAACCTGGGTACACTGTCTATTACCCTATGCTGACTCAGAGTAAAGAGGACGTGGTTGTATGgaaggattgatgacattcgtagtacgtacatcatttcgtgaggctattaaggacatgcacgaagag                                                                                                                                                                                                                                            >  1:319818‑M1/1‑235 (MQ=255)`  
                                                                   `acaAGAAGCGATGCGCTGGGTTGGAGACCACTGGAACTTCCGGTGTCTTCTGGATACCGAAGGTAAGATGGTTCCTAATTGGGCGATTTGCCACTGATACAGGAGGCTACTCATGAACGAAAGACACTTAACAGGTGCTGCTTCTGAAATGCTAGTAGCCTACAAATTTACCAAAGCTGGGTACACTGTCTATTACCCTATGCTGACTCAGAGTAAAGAGGACTGGGTTGTATGgaaggattgatgacattcgtagtacgtacatcatttcgtgaggccattaaggacatgcacgaagagt                                                                                                                                                                                                                                           <  1:186639‑M1/301‑68 (MQ=255)`  
                                                                       `gaagcgaagcGCTGGGTTGGAGACCACTGGAACTTCCGGTGTCTTCTGGATACCGAAGGTAAGAGGGGTCCTAATTGGGCGATTTGCCACTGATACAGGAGGCTACTCATGAACGAAAGACACTTAACAGGTGCTTCTTCTGAAATGCTAGTAGCCTACAAATTTACCAAAGCTGGGTACACTGTCTATTACCCTACGCTGACTCAGAGTAAAGAGGACTTGGTTGTATGgaaggattgaggacattcgtagtacgtacatcatttcgtgaggccattaaggacatgcacgaagagtatgc                                                                                                                                                                                                                                       <  1:84353‑M1/301‑72 (MQ=255)`  
                                                                          `gagatGCGCGGGGTGGGAGACCACTGGAACTTCCGGTGTCTTCTGGATACCGAAGTTAAGATGGGTCCTAATTGGGCGATTTGCCACTGATACAGGAGGCTACTCATGAACGAAAGACACTTAACAGGTGCTGCTTCTGAAATGCTAGTAGCCTACAAATTTACCAAAGCTGGGTACACTGTCTATTACCATATGCTGACTCAGAGTAAAGAGGACTTGGTTGTATGgaaggattgatgacattcgtagtacgtacatcatttcgtgaggccattaaggacatgcacgaagagtatgcaga                                                                                                                                                                                                                                    <  1:204045‑M1/299‑75 (MQ=255)`  
                                                                                   `tGGGTTGGAGACCACTGGAACTTCCGGTGTCTTCTGGATACCGAAGGGAAGATGGGTCCTAATTGGGCGATTTGCCACTGATACAGGAGGCTACTCATGAACGAAAGACACTTAACAGGTGCTGCTTCTGAAATGCTAGTAGCCTACAAATTTACCAAAGCTGGGTACACTGTCTATTACCCTATGCTGACTCAGAGTAAAGAGGACTTGGTTGTATGTAAGGATGGTAAATTTAGTAAGGTTCAGGTTAAAACAGCCACAACGGTTCAAACCAACACAGGAGATGCCAAGCAGGTTAGGc                                                                                                                                                                                                                           <  1:236635/301‑1 (MQ=255)`  
                                                                                   `ggggtTGGAGACCACTGGAACTTCCGGTGTCTTCTGGATACCGAAGGTAAGATGGGTCCTAATTGGGCGATTTGCCACTGATACAGGAGGCTACTCATGAACGAAAGACACTTAACAGGTGCTGCTTCTGAAATGCTAGTAGCCTACAAATTTACCAAAGCTGGGTACACTGTCTATTACCCTATGCTGACTCAGAGTAAAGAGGACTTGGTTGTATGgaaggattgatgacattcgtagtacgtacatcatttcgtgaggccattaaggacatgcacgaagagtatgcagataagaactc                                                                                                                                                                                                                           <  1:275714‑M1/300‑84 (MQ=255)`  
                                                                                               `cACTGGAACTTCCGGTGTCTTCTGGATACCGAAGGTAAGATGGGTCCTAATTGGGCGATTTGCCACTGATACAGGAGGCTATTCATGAACGAAAGACACTTAACAGGTGCTGCTTCTGAAATGCTAGTAGCCTACAAATTTACCAAAGCTCGGTACACTGTCTATTACCCTATGCTTACTCAGAGTACAGCGGACTTGGTTGTATTTAAGGCTTGTAATTTTATTAAGGTTCAGGTTAACTCAGCCACAACGTTTCACACCACCCCATGAGATGCCACGCAGGTCCGGTTAGGTGGAcgcg                                                                                                                                                                                                               >  1:160317/1‑301 (MQ=255)`  
                                                                                                 `cTGGAACTTCCGGTGTCTTCTGGATACCGAAGGTAAGATGGGTCCTAATTGGGCGATTTGCCACTGATACAGGAGGCTACTCATGAACGAAAGCCACTTAACAGGTGCTGCTTCTGAAATGCTAGTAGCCTACAAATTTACCAAAGCTGGTTACACTGTCTATTACCCTATGCGGACTCAAGTTAAAGAGAACTTGTTTGTATGgaaggattgataacattctaagtacgtacatcatttctttagtacattaagaacatgacagaagagtggtcacttataaacttccccaagaccacga                                                                                                                                                                                                             >  1:166393‑M1/1‑204 (MQ=255)`  
                                                                                                   `ggAACTTCCGGTGTCTTCTGGATACCGAAGGTAAGATGGGTCCTAATTGGGCGATTTGCCACTGATACAGGAGGCTACTCATGAACGAAAGACACTTAACAGGTGCTGCTTCTGAAATGCTAGTAGCCTACAAATTTACCAAAGCTGGGTACACTGTCTATTACCCTATGCTGACTCAGAGTAAAGAGGACTTGGTTGTATGTAAGGATGGTAAATTTAGTAAGGTTCAGGTTAAAACAGCCACAACGGTTCAAACCAACACAGGAGATGCCAAGCAGGTTAGGCTAGGTGGATGCGGTAg                                                                                                                                                                                                           >  1:298072/1‑301 (MQ=255)`  
                                                                                                               `gtCTTCTGGATACCGAAGGTAAGATGGGTCCTAATTGGGCGATTTGCCACTGATACAGGAGGCTACTCATGAACGAAAGACACTTAACAGGTGCTGCTTCTGAAATGCTAGTAGCCTACAAATTTACCAAAGCTGGGTACACTGTCTATTACCCTATGCTGACTCAGAGTAAAGAGGACTTGGTTGTATGgaaggattgatgacattcgtagtacgtacatcatttcgtgaggccattaaggacatgcacgaagagtatgcagataaagactctttcaaacaatctcctgcaacagtacgg                                                                                                                                                                                               >  1:280719‑M1/1‑190 (MQ=255)`  
                                                                                                               `gtCTTCTGGATACCGAAGGTAAGATGGGTCCTAATTGGGCGATTTGCCACTGATACAGGAGGCTACTCATGAACGAAAGACACTTAACAGGTGCTGCTTCTGAAATGCTAGTAGCATACAAATTTACCAAAGCTGGGTACACTGTCTATTACCCTATGCTGACTCAGAGTCAAGACGACTTGGTTGTATGTAAGGATGGTAAATTTAGTAAGGTTCAGGTTAAAACAGACACAACGGTTCAAACCAACACAGGAGATGCCAAGCAAGTGAGGCTAGGTGGATGCGGTCGGTGCGAATATaa                                                                                                                                                                                               >  1:334968/1‑301 (MQ=255)`  
                                                                                                                 `gtgtataagagacagggTAAGATGGGTCCTAATTGGGCGATTTGCCACTGATACAGGAGGCTACTCATGAACGAAAGACACTTAACAGGTGCTGCTTCTGAAATGCTAGTAGCCTACAAATTTACCAAAGCTGGGTACACTGTCTATTACCCTATGCTGACTCAGAGTAAAGAGGACTTGGTTGTATGTAAGGATGGTAAATTTAGTAAGGTTCAGGTTAAAACAGCCACAACGGTTCAAACCAACACAGGAGATGCCAAGCAGGTTAGGCTAGGTGGATGCGGTAGGTCCGAATATAAgg                                                                                                                                                                                             <  1:266311/286‑1 (MQ=255)`  
                                                                                                                 `cttctGGATACCGAAGGTAAGATGGGTCCTAATTGGGCGATTTGCCACTGATACAGGAGGCTACTCATGAACGAAAGACACTTAACAGGTGCTGCTTCTGAAATGCTAGTAGCCTACAAATTTACCAAAGCTGGGTACACTGTCTATTACCCTATGCTGACTCAGAGTAAAGAGGACTTGGTTGTATGgaaggattgctgacattcgtagtacgtacatcatttcgtgaggccattaaggacatgcacgaagagtatgcagataaggactctttcaaacaatctcctgcaacagtacggga                                                                                                                                                                                             >  1:30330‑M1/1‑188 (MQ=255)`  
                                                                                                                 `cttctGGATACCGAAGGTAAGATGGGTCCTAATTGGGCGATTTGCCACTGATACAGGAGGCTACTCATGAACGAAAGACACTTAACAGGTGCTGCTTCTGAAATGCTAGTAGCCTACAAATTTACCAAAGCTGGGTACACTGTCTATTACCATATGTTGACTCAGAGTAAAGAGGACTTGGTTGTATGTAAGGATGGTAAATTTAGTAAGGTTCAGGTTAAAACAGCCACAACGGTTCAAACCAACACAGGAGATGCCAAGCAGGTTAGGCTAGGTGGATGCGGTAGGTACGAATATAAgg                                                                                                                                                                                             >  1:76230/1‑301 (MQ=255)`  
                                                                                                                 `cttctGGATACCGAAGGTAAGATGGGTCCTAATTGGGCGATTTGCCACTGATACAGGAGGCTACTCATGAACGAAAGACACTTAACAGATGCTGCTTCTGAAATGCTAGTAGCCTACAAATTTACCAAAGCTGGGTACACTGTCTATTACCCTATGCTGACTCAGAGTAAAGAGGACTTGGTTGTATGgaaggattgatgacattcgtagtacgtacatcatttcgtgaggccattaaggacatgcacgaagagtatgcagataaggactctttcaaacctgtctcttatacacattccag                                                                                                                                                                                             >  1:218797‑M1/1‑188 (MQ=255)`  
                                                                                                                 `cttctGGATACCGAAGGTAAGATGGGTCCTAATTGGGAGATTTGCCACTGATACAGGAGGCTACTCATGAACGAAAGACACTTAACAGGTGCTGCTTCTGAAATGCTAGTAGCCTACAAATTTACCAAAGCTGGGAACACTGTCTATTACCCTATGCTGACTCAGAGTAAAGAGGACTTGGTTGTATGTAAGGATGGTAAATTTAGTAAGGTTCAGGTTAAAACAGCCACAACGGTTCAAACCAACACAGGAGATGCCAAGCAGGTTAGGCTAGGTGGATGCGGTAGGTCCGAATATAAgg                                                                                                                                                                                             >  1:110849/1‑301 (MQ=255)`  
                                                                                                                   `tctGGATACCGAAGGTAAGATGGGTCCTAATTGGGCGATTTGCCACTGATACAGGAGGCTACTCATGAACGAAAGACACTTAACAGGTGCTGCTTCTGAAATGCTAGTAGCCTACAAATTTACCAAAGCTGGGTACACTGTCTATTACCCTATGCTGACTCAGAGTAAAGAGGACTTGGTTGTATGTAAGGATGGTAAATTTAGTAAGGTTCAGGTTAAAACAGCCACAACGGTTCAAACCAACACAGGAGATGCCAAGCAGGTTAGGCTAGGTGGATGCGGTAGGTCCGAATATAAGGAt                                                                                                                                                                                           >  1:212759/1‑301 (MQ=255)`  
                                                                                                                   `agacagtaccgaaGGTAAGATGGGTCCTAATTGGGCGATTTGCCACTGATACAGGAGGCTACTCATGAACGAAAGACACTTAACAGGTGCTGCTTCTGAAATGCTAGTAGCCTACAAATTTACCAAAGCTGGGTACACTGTCTATTACCCTATGCTGACTCAGAGTAAAGAGGACTTGGTTGTATGgaaggattgatgacattcgtagtacgtacatcatttcgtgaggccattaaggacatgcacgaagagtatgcagataaggactctttcaaacaatctcctgcaacagtacgggagg                                                                                                                                                                                           <  1:326986‑M1/295‑116 (MQ=255)`  
                                                                                                                           `gacaggttaagatggATACTAATTGGGCGATTTTCAACTGATACAGGAGGCTACTCATGAACGAAAGACACTTAACAGGTGCTGCTTCTGAAATGCTAGTAGCCTACAAATTTACCAAAGCGGGGTACACTGTCTATTACCCTATGCTGACTCAGAGTAAAGAGGACTTGGTTGTATGgaaggattgatgacattcgtagtacgtaaagcatgtcgtgaggcccttaaggaaatgcacgaagagtatgcagataaggactctttcaaacaatctcctgcaacagtacgggaggtgttctga                                                                                                                                                                                   <  1:340596‑M1/294‑124 (MQ=255)`  
                                                                                                                                `ggTAAGATGGGTCCTAATTGGGCGATTTGCCACTGATACAGGAGGCTACTCATGAACGAAAGACACTTAACAGGTGCTGCTTCTGAAATGCTAGTAGCCTACAAATTTACCAAAGCTGGGTACACTGTCTATTACCCTATGCTGACTCAGAGTAAAGAGGACTTGGTTGTATGgaaggattgatgacattcgtagtacgtacatcatttcgtgaggccattaaggacatgcacgaagagtatgcagataaggactctttcaaacaatctcctgcaacagtacgggaggtgttctgatgtct                                                                                                                                                                              >  1:287368‑M1/1‑173 (MQ=255)`  
                                                                                                                                     `gATGGGTCCTAATTGGGCGATTTGCCACTGATACAGGAGGCTACTCATGAACGAAAGACACTTAACAGGTGCTGCTTCTGAAATGCTAGTAGCCTACAAATTTACCAAAGCTGGGTACACTGTCTATTACCCTATGCTGACTCAGAGTAAAGAGGACTTGGTTGTATGgaaggattgatgacattcgtagtacgtacatcatttcgtgaggccattaaggacatgcacgaagagtatgcagataaggactctttcaaacaatctcctgcaacagtacgggaggtgttctgatgtctgacta                                                                                                                                                                         >  1:346417‑M1/1‑168 (MQ=255)`  
                                                                                                                                     `gATGGGTCCTAATTGGGCGATTTGCCACTGATACAGGAGGCTACTCATGAACGAAAGACACTTAACAGGTGCTGCTTCTGAAATGCTAGTAGCCTACAAATTTAACAAAGCTGGGTACACTGTCTATTACCCTATGCTGACTCAGAGTAAAGAGGACTTGGTTGTATGgaaggattgatgacattcgtagtacgtacatcatttcgtgaggccattaaggacatgcacgaagagtatgcagataaggactctttcaaacaatctcctgcaacagtacgggaggtgttctgatgtctgacta                                                                                                                                                                         >  1:239845‑M1/1‑168 (MQ=255)`  
                                                                                                                                            `ccTAAGTGGGCGATTTGCCACTGATACAGGAGGCTACTCATGAACGAAAGACACTTAACAGGTGCTGCTTCTGAAATGCTAGTAGCCTACAAATTTACCAAAGCTGGGTACACTGTCTATTACCCTATGCTGACTCAGAATAAAGAGGACTTGGTTGTATGTAAGGATGGTAAATTTAGTAAGGTTCAGGTTAAAACAGCCACAACGGTTCAAACCAACACAGGAGATGCCAAGCAGGTTAGGCTAGGTGGATGCGGTAGGTCCGAATATAAGGATGGAGACTTTGACATTCTTGCGGTtg                                                                                                                                                                  <  1:289483/301‑1 (MQ=255)`  
                                                                                                                                             `cTAATTGGGCGATTTGCCACTGATACAGGAGGCTACTCATGAACGAAAGACACTTAACAGGTGCTGCTTCTGAAATGCTAGTAGCCTACAAATTTACCAAAGCTGGGTACACTGTCTATTACCCTATGCTGACTCAGAGTAAAGAGGACTTGGTTGTATGgaaggattgatgacattcgtagtacgtacatcatttcgtgaggccattaaggacatgcacgaagagtatgcagataaggactctttcaaacaatctcctgcaacagtacgggaggtgttctgatgtctgactacctgaaag                                                                                                                                                                 <  1:281932‑M1/301‑142 (MQ=255)`  
                                                                                                                                              `tAATTGGGCGATTTGCCACTGATACAGGAGGCTACTCATGAACGAAAGACACTTAACAGGTGCTGCTTCTGAAATGCTAGTAGCCTACAAATTTACCAAAGCTGGGTACACTGTCTATTACCCTATGCTGACTCAGAGTAAAGAGGACTTGGTTGTATGgaaggattgatgacattcgtagtacgtacatcatttcgtgaggccattaaggacatgcacgaagagtatgcagataaggactctttcaaacaatctcctgcaacagtacgggaggtgttctgatgtctgactacctgaaagt                                                                                                                                                                <  1:108407‑M1/301‑143 (MQ=255)`  
                                                                                                                                                                `agagacagggaggCTACTCATGAACGAAAGACACTTAACAGGTGCTGCTTCTGAAATGCTAGTAGCCTACAAATTTACCAAAGCTGGGTACACTGTCTATTACCCTATGCTGACTCAGAGTAAAGAGGACTTGGTTGTATGTAAGGATGGTAAATTTAGTAAGGTTCAGGTTAAAACAGCCACAACGGTTCAAACCAACACAGGAGATGCCAAGCAGGTTAGGCTAGGTGGATGCGGTAGGTCCGAATATAAGGATGGAGACTTTGACATTCTTGCGGTTGTGGTTGACGAAGATGTGCtt                                                                                                                                              <  1:277553/293‑1 (MQ=255)`  
                                                                                                                                                                      `cAGGAGGCTACTCATGAACGAAAGACACTTAACAGGTGCTGCTTCTGAAATGCTAGTAGCCTACAAATTTACCAAAGCTGGGTACACTGTCTATTACCCTATGCTGACTCAGAGTAAAGAGGACTTGGTTGTATGTAAGGATGGTAAATTTAGTAAGGTTCAGGTTAAAACAGCCACAACGGTTCAAACCAACACAGGAGATGCCAAGCAGGTTAGGCTAGGTGGATGCGGTAGGTCCGAATATAAGGATGGAGACTTTGACATTCTTGCGGTTGTGGTTGACGAAGATGTGCTTATTTTc                                                                                                                                        >  1:153519/1‑301 (MQ=255)`  
                                                                                                                                                                           `ggCTACTCATGAACGAAAGACACTTAACAGGTGCTGCTTCTGAAATGCTAGTAGCCTACAAATTTACCAAAGCTGGGTACACTGTCTATTACCCTATGCTGACTCAGAGTAAAGAGGACTTGGTTGTATGgaaggattgatgacattcgtagtacgtacatcatttcgtgaggccattaaggacatgcacgaagagtatgcagataaggactctttcaaacaatctcctgcaacagtacgggaggtgttctgatgtctgactacctgaaagtgctgcaagcaatcaaaagttgccctaaga                                                                                                                                   >  1:135900‑M1/1‑130 (MQ=255)`  
                                                                                                                                                                                         `aaaaGACACTTAACAGGTGCTGCTTCTGAAATGCTAGTAGCCTACAAATTTACCAAAGCTGGGTACACTGTCTATTACCCTATGCTGACTCAGAGTAAAGAGGACTTGGTTGTATGTAAGGATGGTAAATTTAGTAAGGTTCAGGTTAAAACAGCCACAACGGTTCAAACCAACACAGGAGATGCCAAGCAGGTTAGGCTAGGTGGATGCGGTAGGTCCGAATATAAGGATGGAGACTTTGACATTCTTGCGGTTGTGGTTGACGAAGATGTGCTTATTTTCACATGGGACGAAGTAAAAg                                                                                                                     <  1:39495/300‑1 (MQ=255)`  
                                                                                                                                                                                           `aaGACACTTAACAGGTGCTGCTTCTGAAATGCTAGTAGCCTACAAATTTACCAAAGCTGGGTACACTGTCTATTACCCTATGCTGACTCAGAGTAAAGAGGACTTGGTTGTATGgaaggattgatgacattcgtagtacgtacatcatttcgtgaggccattaaggacatgcacgaagagtatgcagataaggactctttcaaacaatctcctgcaacagtacgggaggtgttctgatgtctgactacctgaaagtgctgcaagcaatcaatagttgccctaagactttccagtccaacta                                                                                                                   <  1:133222‑M1/301‑188 (MQ=255)`  
                                                                                                                                                                                           `aaGACACTTAACAGGTGCTGCTTCTGAAATGCTAGTAGCCTACAAATTTACCAAAGCTGGGTACACTGTCTATTACCCTATGCTGACTCAGAGTAAAGAGGACTTGGTTGTATGgaaggattgatgacattcgtagtacgtacatcatttcgtgaggccattaaggacatgcacgaagagtatgcagataaggactctttcaaacaatctcctgcaacagtacgggaggtgttctgatgtctgactacctgaaagtgctgcaagcaatcaaaagttgccctaagactttccagtccaacta                                                                                                                   <  1:273276‑M1/301‑188 (MQ=255)`  
                                                                                                                                                                                           `aaGACACGTAACAGGTGCTGCTTCTGAAATGCTAGTAGCCTACAAATTTACCAAAGCTGGGTACACTGTCTATTACCCTATGATGACTCAGAATAAAGAGGACTTGGTTGTATGTAAGGATGGTAAATTTAGTAAGGTTCAGGTTAAAACAGCCACAACGGTTCAAGCCAACCAAGGAGATGCCAAGCAGGTTAGGCTAGGTGGATTCGGTAGGTCCGAATATAAGGACGGAGACTTTTACATTCTTGCGGTTGTGGTTGACGAAGATGTGCTTATTTTCACATGGGACGAAGTAAAAGGt                                                                                                                   <  1:120475/301‑1 (MQ=255)`  
                                                                                                                                                                                                `acTTAACAGGTGCTGCTTCTGAAATGCTAGTAGCCTACAAATTTACCAAAGCTGGGTACACTGTCTATTACCCTATGCTGACTCAGAGTAAAGAGGACTTGGTTGTATGgaaggattgatgacattcgtagtacgtacatcatttcgtgaggccattaaggacatgcacgaagagtatgcagataaggactctttcaaacaatctcctgcaacagtacgggaggtgttctgatgtctgactacctgaaagtgctgcaagcaatcaaaagttgccctaagactttccagtccaactatgtac                                                                                                              <  1:4251‑M1/301‑193 (MQ=255)`  
                                                                                                                                                                                                 `cTTAACAGGTGCTGCTTCTGAAATGCTAGTAGCCTACAAATTTACCAAAGCTGGGTACACTGTCTATTACCCTATGCTGACTCAGAGTAAAGAGGACTTGGTTGTATGgaaggattgatgacattcgtagtacgtacatcatttcgtgaggccattaaggacatgcacgaagagtatgcagataaggactctttcaaacaatctcctgcaacagtacgggaggtgttctgatgtctgactacctgaaagtgctgcaagcaatcaaaagttgccctaagactttccagtcctgtctcttata                                                                                                             >  1:205645‑M1/1‑108 (MQ=255)`  
                                                                                                                                                                                                 `cTTAACAGGTGCTGCTTCTGAAATGCTAGTAGCCTACAAATTTACCAAAGCTGGGTACACTGTCTATTACCCTATGCTGACTCAGAGTAAAGAGGACTTGGTTGTATGgaaggattgatgacattcgtagtacgtacatcatttcgtgaggccattaaggacatgcacgaagagtatgcagataaggactctttcaaacaatctcctgcaacagtacgggaggtgttctgatgtctgactacctgaaagtgctgcaagcaatcaaaagttgccctaagactttccagtccaactatgtacg                                                                                                             >  1:157144‑M1/1‑108 (MQ=255)`  
                                                                                                                                                                                                 `cTTAACAGGTGCTGCTTCTGAAATGCTAGTAGCCTACAAATTTACCAAAGCTGGGTACACTGTCTATTACCCTATGCTGACTCAGAGTAAAGAGGACTTGGTTGTATGgaaggattgatgacattcgtagtacgtacatcatttcgtgaggccattaaggacatgcacgaagagtatgcagataaggactctttcaaacaatctcctgcaacagtacgggaggtgttctgatgtctgactacctgaaagtgctgcaagcaatcaaaagttgccctaagactttccagtccaacgatgtacg                                                                                                             >  1:44818‑M1/1‑108 (MQ=255)`  
                                                                                                                                                                                                 `cTTAACAGGTGCTGCTTCTGAAATGCTAGTAGCCTACAAATTTACCAAAGCTGGGTACACTGTCTATTACCCTATGCTGACTCAGAGTAAAGAGGACTTGGTTGTATGTAAGGATGGTAAATTTAGTAAGGTTCAGGTTAAAACAGCCACAACGGTTCAAACCAACACAGGAGATGCCAAGCAGGTTAGGCTAGGTGGATGCGGTAGGTCCGAATATAAGGATGGAGACTTTGACATTCTTGCGGTTGTGGTTGACGAAGATGTGCTTATTTTCACATGGGACGAAGTAAAAGGTAAGACa                                                                                                             >  1:181583/1‑301 (MQ=255)`  
                                                                                                                                                                                                     `aCAGGTGCTGCTTCTGAAATGCTAGTAGCCTACAAATTTACCAAAGCTGGGTACACTGTCTATTACCCTATGCTGACTCAGAGTAAAGAGGACTTGGTTGTATGgaaggattgatgacattcgtagtacgtacatcatttcgtgaggccattaaggacatgcacgaagagtatgcagacaaggactctttcaaacaatctcctgcaacagtacgggaggtgttctgatgtctgactacctgaaagtgctgcaagcaatcaaaagttgccctaagactttccagtccaactatgtacggaac                                                                                                         <  1:3712‑M1/301‑198 (MQ=255)`  
                                                                                                                                                                                                     `aCAGGTGCCGCTTCTGAAATGCTAGTAGCCTACAAATTTACCAAAGCTGGGTACACTGTCTATTACCCTATGCTGACTCAGAGTAAAGAGGACTTGGTTGTATGgaaggattgatgacattcgtagtacgtacatcatttcgtgaggccattaaggacatgcacgaagagtatgcagataaggactcttttaaacaatctcctgcaacagtacgggaggtgttctgatgtctgactacctgaaagtgctgcaagcaatcaaaagttgccctaagactttccagtccaactatgtacggaac                                                                                                         <  1:89217‑M1/301‑198 (MQ=255)`  
                                                                                                                                                                                                      `cAGGTGCTGCTTCTGAAATGCTAGTAGCCTACAAATTTACCAAAGCTGGGTACACTGTCTATTACCCTATGCTGACTCAGAGTAAAGAGGACTTGGTTGTATGTAAGGATGGTAAATTTAGTAAGGTTCAGGTTAAAACAGCCACAACGGTTCAAACCAACACAGGAGATGCCAAGCAGGTTAGGCTAGGTGGATGCGGTAGGTCCGAATATAAGGATGGAGACTTTGACATTCTTGCGGTTGTGGTTGACGAAGATGTGCTTATTTTCACATGGGACGAAGTAAAAGGTAAGACATCCAt                                                                                                        >  1:293831/1‑301 (MQ=255)`  
                                                                                                                                                                                                         `tttcttctTCTGAAATGCTAGTAGCCTACAAATTTACCAAAGCTGGGTACACTGTCTATTACCCTATGCTGACTCAGAGTAAAGAGGACTTGGTTGTATGTAAGGATGGTAAATTTAGTAAGGTTCAGGTTAAAACAGCCACAACGGTTCAAACCAACACAGGAGATGCCAAGCAGGTTAGGGTAGGTGGATGCGGTAGGTCCGAATATAAGGATGGAGACTTTGACATTCTTGCGGTTGTGGTTGACGAAGATGTGCTTATTTTCACATGGGACGAAGTAAAAGGTAAGACATCCAtgtg                                                                                                     <  1:225183/295‑1 (MQ=255)`  
                                                                                                                                                                                                                `agctgAAATGCTAGTAGCCTACAAATTTACCAAAGCTGGGTACACTGTCTATTACCCTATGCTGACTCAGAGTAAAGAGGACTTGGTTGTATGgaaggattgatgacattcgtagtacgtacatcatttcgtgaggccattaaggacatgcacgaagagtatgcagataaggactctttcaaacaatctcctgcaacagtacgggaggtgttctgatgtctgactacctgaaagtgctgcaagcaatcaaaagttgccctaagactttccagtccaactatgtacggaacaatgcgagcct                                                                                              <  1:56622‑M1/299‑209 (MQ=255)`  
                                                                                                                                                                                                                 `tCTGAAATGCTAGTAGCCTACAAATTTACCAAAGCTGGGTACACTGTCTATTACCCTATGCTGACTCAGAGTAAAGAGGACTTGGTTGTATGgaaggattgatgacattcgtagtacgtacatcatttcgtgaggccattaaggacatgcacgaagagtatgcagataaggactctttcaaacaatctcctgcaacagtacgggaggtgttctgatgtctgactacctgaaagtgctgcaagcaatcaaaagttgccctaagactttccagtccaactatgtacggaacaatgcgagcctc                                                                                             >  1:247940‑M1/1‑92 (MQ=255)`  
                                                                                                                                                                                                                        `tGCTAGTAGCCTACAAATTTACCAAAGCTGGGTACACTGTCTATTACCCTATGCTGACTCAGAGTAAAGAGGACTTGGTTGTATGTAAGGATGGTAAATTTAGTAAGGTTCAGGTTAAAACAGCCACAACGGTTCAAACCAACACAGGAGATGCCAAGCAGGTTAGGCTAGGTGGATGCGGTAGGTCCGAATATAAGGATGGAGACTTTGACATTCTTGCGGTTGTGGTTGACGAAGATGTGCTTATTTTCACATGGGACGAAGTAAAAGGTAAGACATCCATGTGTGTCGGCAAGAGaaa                                                                                      >  1:2079/1‑301 (MQ=255)`  
                                                                                                                                                                                                                              `tagCCTACAAATTTACCAAAGCTGGGTACACTGTCTATTACCCTATGCTGACTCAGAGTAAAGAGGACTTGGTTGTATGgaaggattgatgacattcgtagtacgtacatcatttcgtgaggccattaaggacatgcacgaagagtatgcagataaggactctttcaaacaatctcctgcaacagtacgggaggtgttctgatgtctgactacctgaaagtgctgcaagcaatcaaaagttgccctaagactttccagtccaactatgtacggaacaatgcgagcctcgtagcggaggccg                                                                                <  1:233807‑M1/301‑223 (MQ=255)`  
                                                                                                                                                                                                                                       `aaTTTACCAAAGCTGGGTACACTGTCTATTACCCTATGCTGACTCAGAGTAAAGAGGACTTGGTTGTATGgaaggattgatgacattcgtagtacgtacatcatttcgtgaggccattaaggacatgcacgaagagtatgcagataaggactctttcaaacaatctcctgcaacagtacgggaggtgttctgatgtctgactacctgaaagtgctgcaagcaatcaaaagttgccctaagactttccagtccaactatgtacggaacaatgcgagcctcgtagcggaggccgcttcccgtg                                                                       <  1:60460‑M1/301‑232 (MQ=255)`  
                                                                                                                                                                                                                                                `aaGCTGGGTACACTGTCGATTACCCTATGCTGACTCAGAGTAAAGAGGACTTGGTTGTATGgaaggattgatgacattcgtagtacgtacatcatttcgtgaggccattaaggacatgcacgaagagtatgcagataaggactctttcaaacaatctcctgcaacagtacgggaggtgttctgatgtctgactacctgaaagtgctgcaagcaatcaaaagttgccctaagactttccagtccaactatgtacggaacaatgcgagcctcgtagcggaggccgcttcccgtggtcacatct                                                              <  1:65240‑M1/301‑241 (MQ=255)`  
                                                                                                                                                                                                                                                          `cacTGTCTATTACCCTATGCTGACTCAGAGTAAAGAGGACTTGGTTGTATGTAAGGATGGTAAATTTAGTAAGGTTCAGGTTAAAACAGCCACAACGGTTCAAACCAACACAGGAGATGCCAAGCAGGTTAGGCTAGGTGGATGCGGTTGGTCCGTATATAAGGATGGAGACTTTGACATTCTTGCGGTTGTGGTTGACGAAGATGTTCTTATTTTCACATGGGACGAAGTAAAATGTAAGACATCCATGTGTGTCGGCAAGAGAAACAAAGGCATCAAACTATAGGAGGAatcctgtccc                                                    >  1:192859/1‑293 (MQ=255)`  
                                                                                                                                                                                                                                                           `aaggTCTATTACCCGATGCTGACTCAGAGTAAAGAGGACTTGGTTGTATGTAAGGATGGTAAATTTAGTAAGGTTCAGGTTAAAACAGCCACAACGGTTCAAACCAACACAGGAGATGCCAAGCAGGTTAGGCTAGGTGGATGCGGTAGGTCCGAATATAAGGATGGAGACTTTGACATTCTTGCGGTTGTGGTTGACGAAGATGTGCTTATTTTCACATGGGACGAAGTAAAAGGTAAGACATCCATGTGTGTCGGCAAGAGAAACAAAGGCATAAAACTATAGGAGAAATTATTAGGGc                                                   <  1:189874/298‑1 (MQ=255)`  
                                                                                                                                                                                                                                                              `gTCTATTACCCTATGCTGACTCAGAGTAAAGAGGACTTGGTTGTATGgaaggattgatgacattcgtagtacgtacatcatttcgtgaggccattaaggacatgcacgaagagtatgcagataaggactctttaaaacaatctcctgcaacagtacgggaggtgttctgatgtctgactacctgaaagtgctgcaagcaatcaaaagttgccctaagactttccagtccaactatgtacggaacaatgcgagcctcgtaggggaggacgcttcccgtggtcacatctcggtcccgactaat                                                >  1:53662‑M1/1‑47 (MQ=255)`  
                                                                                                                                                                                                                                                              `gTCTATTACCCTATGCTGACTCAGAGTAAAGAGGACTTGGTTGTATGTAAGGATGGTAACTTTAGTAAGGTTCAGGTTAAAACAGCCACAACGGTTCAAACCAACACAGGAGATGCCAAGCAGGTTAGGCTAGGTGGATGCGGTAGGTCCGAATATAAGGATGGAGACTTTGACATTCTTGCGGTTGTGGTTGACGAAGATGTGCTTATTTTCACATGGGACGAAGTAAAAGGTAAGACATCCATGTGTGTCGGCAAGAGAAACAAAGGCATAAAACTATAGGAGAAATTATTATGGCTAt                                                >  1:338485/1‑301 (MQ=255)`  
                                                                                                                                                                                                                                                              `gTCTATTACCCTATGCTGACTCAGAGTAAAGAGGACTTGGTTGTATGTAAGGATGGTAAATTTAGTACGGTTCTTCTTAAAACAGCCTCTTCGGTTCAAACCTACCCAGGAGATTCCTTTCAGGTTATTCTTGTTGGATGCTTTCTTTCCGAATATATTTTTGGAGACTTTCCTCTTCTTCCGTTTTTTCTTGACGAAGATGTGCTTATTTTCACATGGTACGTATTTTTCTGTAAGACATCCATGTGTTTCGTTAAGAGAATCATAGCCATAAACCTATACGACAAattctgtctctttt                                                >  1:201850/1‑290 (MQ=255)`  
                                                                                                                                                                                                                                                              `gTCTATTACCCTATGCTGACTCAGAGTAAAGAGGACTTGGTTGTATGTAAGGATGGTAAATTTAGTAAGGTTCAGGTTAAAACAGCCACAACGGTTCAAACCAACACAGGAGATGCCAAGCAGGTTAGGCTAGGTGGATGCGGTAGGTCCGAATATAAGGATGGAGACTTTGACATTCTTGCGGTTGTGGTTGACGAAGATGTGCTTATTTTCACATGGGACGAAGTAAAAGGTAAGACATCCATGTGTTTCGGCAAGAGAAACAAAGGCATAAAACTATAGGAGAAAttattactgtctc                                                >  1:33047/1‑294 (MQ=255)`  
                                                                                                                                                                                                                                                              `gTCTATTACCCTATGCTGACTCAGAGTAAAGAGGACTTGGTTGTATGTAAGGATGGTAAATTTAGTAAGGTTCAGGTTAAAACAGCCACAACGGTTCAAACCAACACAGGAGATGCCAAGCAGGTTAGGCTAGGTGGATGCGGTAGGTCCGAATATAAGGATGGAGACTTTGACATTCTTGCGGTTGTGGTTGACGAAGATGTGCTTATTTTCACATGGGACGAAGTAAAAGGTAAGACATCCATGTGTGTCGGCAAGAGAAACAAAGGCATAAAACTATAGGAGAAATTATTATGGCTAt                                                >  1:129762/1‑301 (MQ=255)`  
                                                                                                                                                                                                                                                              `gTCTATTACCCTATGCTGACTCAGAGTAAAGAGGACTTGGTTGTATGTAAGGATGGTAAATTTAGTAAGGTTCAGGTTAAAACAGCCACAACGGTTCAAACCAACACAGGAGATGCCAAGCAGGTTAGGCTAGGTGGATGCGGTAGGTCCGAATATAAGGATGGAGACTTTGACATTCTTGCGGTTGTGGTTGACGAAGATGTGCTTATTTTCACATGGGACGAAGTAAAAGGTAAGACATCCATGTGTGTCGGCAAGAGAAACAAAGGCATAAAACTATAGGAGAAATTATTATGGCTAt                                                >  1:175839/1‑301 (MQ=255)`  
                                                                                                                                                                                                                                                                `cTATTACCCTATGCTGACTCAGAGTAAAGAGGACTTGGTTGTATGgaaggattgctgacattcgtagtacgtacatcatttcttgaggccattaaggacatgcacgaagagtatgcagataaggactctttcaaacaatctcctgcaacagtacgggaggtgttctgatgtctgactacctgaaagttctgcaagcaatcaaaagttgcactaagactttccagtccaactatgtacggaacaatgcgagcctcgtagcggaggccgcttcccgtggtcaactctatttcctgactactgg                                              >  1:155516‑M1/1‑45 (MQ=255)`  
                                                                                                                                                                                                                                                                `cTATTACCCTATGCTGACTCAGAGTAAAGAGGACTTGGTTGTATGgaaggattgatgacattcgtagtacgtacatcatttcgtgaggccattaaggacatgcacgaagagtatgcagataaggactctttcaaacaatctcctgcaacagtacgggaggtgttctgatgtctgactacctgaaagtgctgcaagcaatcaaaagttgccctaagactttccagtccaactatgtacggaacaatgcgagcctcgtagcggaggccgcttcccgtggtcacatctcgtgcctgactactag                                              >  1:123788‑M1/1‑45 (MQ=255)`  
                                                                                                                                                                                                                                                                `cTATTACCCTATGCTGACTCAGAGTAAAGAGGACTTGGTTGTATGgaaggattgatgacattcgtagtacgtacatcatttcgtgaggccattaaggacatgcacgaagagtatgcagataaggactctttcaaacaatctcctgcaacagtacgggaggtgttctgatgtctgactacctgaaagtgctgcaagcaatcaaaagttgccctaagactttccagtccaactatgtacggaacaatgcgagcctcgtagcggaggccgcttcccgtggtcacatctcgtgcctgactactag                                              <  1:241332‑M1/301‑257 (MQ=255)`  
                                                                                                                                                                                                                                                                 `tATTACCCTTTGCTGACTCAGAGTAAAGAGGACTTGGTTGTATGTAAGGATGGTAAATTTAGTAAGGTTCAGGTTAAAACAGCCACAACGGTTCAAACCAACACAGGAGATGCCAAGCAGGTTAGGCTAGGTGGATGCGGTAGGTCCGAATATAAGGATGGAGACTTTGACATTCTTGCGGTTGTGGTTGACGAAGATGTGCTTATTTTCACATGGGACGAAGTAAAAGGTAAGACATCCATGTGTGTCGGCAAGAGAAACAAAGGCATAAAACTATAGGAGAAATTATTATGGCTATGAc                                             <  1:324004/301‑1 (MQ=255)`  
                                                                                                                                                                                                                                                                  `agatgtgtataagagacaggagtaaagaggacttggttgtaTGgaaggattgatgacattcgtagtacgtacatcatttcgtgaggccattaaggacatgcacgaagagtatgcagataaggactctttcaaacaatctcctgcaacagtacgggaggtgttctgatgtctgactacctgaaagtgctgcaagcaatcaaaagttgccctaagactttccagtccaactatgtacggaacaatgcgagcctcgtagcggaggccgcttcccgtggtcacatctcgtgtctgactactagtg                                            <  1:199340‑M1/282‑259 (MQ=255)`  
                                                                                                                                                                                                                                                                  `aTTACCCTATGCTGACTCAGAGTAAAGAGGACTTGGTTGTATGgaaggattgatgacattcgtagtacgtacatcatttcgtgaggccattaaggacatgcacgaagagtatgcagataaggactctttcaaacaatctcctgcaacagtacgggaggtgttctgatgtctgactacctgaaagtgctgcaagcaatcaaaagttgccctaagactttccagtccaactatgtacggaacaatgcgagcctcgtagcggaggccgcttcccgtggtcacatctcgtgcctgactactagtg                                            <  1:163677‑M1/301‑259 (MQ=255)`  
                                                                                                                                                                                                                                                                  `aTTACCCTATGCTGACTCAGAGTAAAGAGGACTTGGTTGTATGgaaggattgatgacattcgtagtacgtacatcatttcgtgaggccattaaggacatgcacgaagagtatgcagataaggactctttcaaacaatctcctgcaacagtacgggaggtgttctgatgtctgactacctgaaagtgctgcaagcaatcaaaagttgccctaagactttccagtccaactatgtacggaacaatgcgagcctcgtagcggaggccgcttcccgtggtcacatctcgtgcctgactactagtg                                            >  1:114075‑M1/1‑43 (MQ=255)`  
                                                                                                                                                                                                                                                                  `aTTACCCTATGCTGACTCAGAGTAAAGAGGACTTGGTTGTATGgaaggattgatgacattcgtagtacgtacatcatttcgtgaggccattaaggacatgcacgaagagtatgcagataaggactctttcaaacaatctcctgcaacagtacgggaggtgttctgatgtctgactacctgaaagtgctgcaagcaatcaaaagttgccctaagactttccagtccaactatgtacggaacaatgcgagcctcgtagcggaggccgcttcccgtggtcacatctcgtgcctgactactagtg                                            <  1:30995‑M1/301‑259 (MQ=255)`  
                                                                                                                                                                                                                                                                    `agacagatgctgaCTCAGAGTAAAGAGGACTTGGTTGTATGgaaggattgatgacattcgtagtacgtacatcatttcgtgaggccattaaggacatgcacgaagagtatgcagataaggactctttcaaacaatctcctgcgacagtacgggaggtgttctgatgtctgactacctgaaagtgctgcaagcaatcaaaagttgccctaagactttccagtccaactatgtacggaacaatgcgagcctcgtagcggaggccgcttcccgtggtcacatctcgtgcctgactactagtgga                                          <  1:81504‑M1/295‑261 (MQ=255)`  
                                                                                                                                                                                                                                                                     `aCCCTATGCTGACTCAGAGTAAAGAGGACTTGGTTGTATGgaaggattgatgacattcgtagtacgtacatcatttcgtgaggccattaaggacatgcacgaagagtatgcagataaggactctttcaaacaatctcctgcaacagtacgggaggtgttctgatgtctgactacctgaaagtgctgcaagcaatcaaaagttgccctaagactttccagtccaactatgtacggaacaatgcgagcctcgtagcggaggccgcttcccgtggctgtctcttatacacatctccgagcccac                                         >  1:249989‑M1/1‑40 (MQ=255)`  
                                                                                                                                                                                                                                                                      `cccTATGCTGACTCAGAGTAAAGAGGACTGGGTTGTAGGTAAGGATGGTAAATTTAGTAAGGTTAAGGTTAAAACAGCCAAAACGGTTCAAACCAACACAGGAGATGCCAAGCAGGTTAGGCTAGGTGGATGCGGTAGGTCCGAATATAAGGATGGAGACTTTGACATTCTTGCGGTTGTGGTTGACGAAGATGTGCTTATTTTCACATGGGACGAAGTAAAAGGTAAGACATCCATGTGTGTCGGCAAGAGAAACAAAGGCATAAAACTATAGGAGAAATTATTATGGCTATGACAAAGa                                        <  1:221759/301‑1 (MQ=255)`  
                                                                                                                                                                                                                                                                       `tgtataagagacaggagtaaagaggacttggTTGTATGgaaggattgatgacattcgtagtacgtacatcatttcgtgaggccattaaggacatgcacgaagagtatgcagataaggactctttcaaacaatctcctgcaacagtacgggaggtgttctgatgtctgactacctgaaagtgctgcaagcaatcaaaagttgccctaagactttccagtccaactatgtacggaacaatgcgagcctcgtagcggaggccgcttcccgtggtcacatctcgtgcctgactactagtggacgt                                       <  1:33190‑M1/287‑264 (MQ=255)`  
                                                                                                                                                                                                                                                                                 `cTCAGAGTAAAGAGGACTTGGTTGTATGTAAGGATGGTAAATTTAGTAAGGTTCAGGTTAAAACAGCCACAACGGTTCAAACCAACACAGGAGATGCCAAGCAGGTTAGGCTAGGTGGATGCGGTAGGTCCGAATATAAGGATGGAGACTTTCACATTCTTGCGGTTGTGGTTGACGAAGATGTGCTTATTTTCACATGGGACGAAGTAAAAGGTAAGACATCCATGTGTGTCGGCAAGAGAAACAAAGGCATAAAACTATAGGAGAAATTATTATGGCTATGACAAAGAAATTTAAAgtg                             <  1:313364/301‑1 (MQ=255)`  
                                                                                                                                                                                                                                                                                  `tCAGAGTAAAGAGGACTTGGTTGTATGgaaggattgatgacattcgtagtacgtacatcatttcgtgaggccattaaggacatgcacgaagagtatgcagataaggactctttcaaacaatctcctgcaacagtacgggaggtgttctgatgtctgactacctgaaagtgctgcaagcaatcaaaagttgccctaagactttccagtccaactatgtacggaacaatgcgagcctcgtatcggaggccgcttcccgtggtcacatctcgtgcctgactactagtggacgtaacggtggcgc                            <  1:300207‑M1/301‑275 (MQ=255)`  
                                                                                                                                                                                                                                                                                     `gagTAAAGAGGACTTGGTTGTATGTAAGGATGGTAAATTTAGTAAGGTTCAGGTTAAAACAGCCACAACGGTTCAAACCAACACAGGAGATGCCAAGCAGGTTAGGCTAGGTGGATGCGGTAGGTCCGAATATAAGGATGGAGACTTTGACATTCTTGCGGTTGTGGTTGACGAAGATGTGCTTATTTTCACATGGGACGAAGTAAAAGGTAAGACATACATGTGTGTCGGCAAGAGCAACAAAGGCATAAAACTATAGGCGAAATTCTTATGGCTATGACAAAGAAATTTAAAGTGTCCt                         >  1:317120/1‑301 (MQ=255)`  
                                                                                                                                                                                                                                                                                         `aaaGAGGACTTGGTTGTATGgaaggattgatgacattcgtagtacgtacatcatttcgtgaggccattaaggacatgcacgaagagtatgcagataaggactctttcaaacaatctcctgcaacagtacgggaggtgttctgatgtctgactacctgaaagtgctgcaagcaatcaaaagttgccctaagactttccagtccaactatgtacggaacaatgcgagcctcgtagcggaggccgcttcccgtggtcacatctcgtgcctgactactagtggacgtaacggtggcgcttgggaa                     >  1:72168‑M1/1‑20 (MQ=255)`  
                                                                                                                                                                                                                                                                                                    `ggTTGTATGgaaggattgatgacattcgtagtacgtacatcatttcgtgaggccattaaggacatgcacgaagagtatgcagataaggactctttcaaacaatctcctgcaacagtacgggaggtgttctgatgtctgactacctgaaagtgctgcaagcaatcaaaagttgccctaagactttccagtccaactatgtacggaacaatgcgagcctcgtagcggaggccgcttcccgtggtcacatctcgtgcctgactactcgtggacgtaacggtggcgcttgggaaatcactgcttc          >  1:51892‑M1/1‑9 (MQ=255)`  
                                                                                                                                                                                                                                                                                                     `gTTGTATGgaaggattgatgacattcgtagtacgtacatcatttcgtgaggccattaaggacatgcacgaagagtatgcagataaggactctttcaaacaatctcctgcaacagtacgggaggtgttctgatgtctgactacctgaaagtgctgcaagcaatcaaaagttgccctaagactttccagtccaactatgtacggaacaatgcgagcctcgtagcggaggccgcttcccgtggtcacatctcgtgcctgactactagtggacgtaacggtggcgcttgggaaatcactgcttcc         >  1:215332‑M1/1‑8 (MQ=255)`  
                                                                                                                                                                                                                                                                                                        `gTATGgaaggattgatgacattcgtagtacgtacatcatttcgtgaggccattaaggacatgcacgaagagtatgcagataaggactctttcaaacaatctcctgcaacagtacgggaggtgttctgatgtctgactacctgaaagtgctgcaagcaatcaaaagttgccctaagactttccagtccaactatgtacggaacaatgcgagcctcgtagcggaggccgcttcccgtggtcacatctcgtgcctgactactagtggacgtaacggtggcgcttgggaaatcactgcttccggt      >  1:26123‑M1/1‑5 (MQ=255)`  
                                                                                                                                                                                                                                                                                                        `gTATGgaaggattgatgacattcgtagtacgtacatcatttcgtgaggccattaaggacatgcacgaagagtatgcagataaggactctttcaaacaatctcctgcaacagtacgggaggtgttatgctgtctgactacctgaaagggctgcaagcaatcaaaagttgccataagactttccagtccaactatatacggaacaatgcgagcctcgtagcggaggccgcttcccgtggtcacatctcgtgcctgactattagtggacgtaacggtgggggttgggaactcactgcttcgggg      >  1:229541‑M1/1‑5 (MQ=255)`  
                                                                                                                                                                                                                                                                                                          `atgtaAGGATGGTAAATTTAGTAAGGTTCAGGTTAAAACAGCCACAACGGTTCAACCCAACACAGGAGATGCCAAGCAGGTTAGGCTAGGTGGATGCGGTAGGTCCGAATATAAGGATGGAGACTTTGACATTCTTGCGGTTGTGGTTGACGAAGATGTGCTTATTTTCACATGGGACGAAGTAAAAGGTAAGACATCCATGTGTGTCGGCAAGAGAAACAAAGGCATAAAACTATAGGAGAAATTATTATGGCTATGACAAAGAAATTTAAAGTGTCCTTCGACGTTACCGCACAGATgt    >  1:142266/1‑301 (MQ=255)`  
                                                                                                                                                                                                                                                                                                          `atgtaAGGATGGTAAATTTAGTAAGGTTCAGGTTAAAACAGCCACAACGGTTCAAACCAACACAGGAGATGCCAAGCAGGTTAGGCTAGGTGGATGCGGTAGGTCCGAATATAAGGATGGAGACTTTGACATTCTTGCGGTTGTGGTTGACGAAGATGTGCTTATTTTCACATGGGACGAAGTAAAAGGTAAGACATCCAAGTGTGTCGGCAAGAGAAACAAAGGCATAAAACTATAGGAGAAATTATTATGGCTATGACAAAGAAATTTAAAGTGTCCTTCGACGTTACCGCAAAGATgt    >  1:79657/1‑301 (MQ=255)`  
                                                                                                                                                                                                                                                                                                          `aggtaAGGATGGTAAATTTAGTAAGGTTCAGGTTAAAACAGCCACAACGGTTCAAACCAACACAGGAGATGCCAAGCAGGTTAGGCTAGGTGGATGCGGTAGGTCCGAATATAAGGATGGAGACTTTGACATTCTTGCGGTTGTGGTTGACGAAGATGTGCTTATTTTCACATGGGACGAAGTAAAAGGTAAGACATCCATGTGTGTCGGCAAGAGAAACAAAGGCATAAAACTATAGGAGAAATTATTATGGCTATGACAAAGAAATTTAAAGTGTCCTTCGACGTTACCGCAAAGATgt    <  1:138277/299‑1 (MQ=255)`  
                                                                                                                                                                                                                                                                                                          `aTGgaaggattgatgacattcgtagtacgtacatcatttcgtgaggccattaaggacatgcacgaagagtatgcagataaggactctttcaaacaatctcctgcaacagtacgggaggtgttctgatgtctgactacctgaaagtgctgcaagcaatcaaaagttgccctaagactttccagtccaactatgtacggaacaatgcgagcctcgtagcggaggccgcttcccgtggtcacatctcgtgcctgactactagtggacgtaacggtggcgcttgggaaatcactgcttccggtac    <  1:179910‑M1/301‑299 (MQ=255)`  

                                                                                                                                                                                                                                                                                                            `|`                                                                                                                                                                                                                                                                                                              
`GGGTACATGATGAAATCCAAGTAGGCTGCCGTACCGAAGAGATTGCTCAGGTGGTCATTGAGACCGCACAAGAAGCGATGCGCTGGGTTGGAGACCACTGGAACTTCCGGTGTCTTCTGGATACCGAAGGTAAGATGGGTCCTAATTGGGCGATTTGCCACTGATACAGGAGGCTACTCATGAACGAAAGACACTTAACAGGTGCTGCTTCTGAAATGCTAGTAGCCTACAAATTTACCAAAGCTGGGTACACTGTCTATTACCCTATGCTGACTCAGAGTAAAGAGGACTTGGTTGTATGTAAGGATGGTAAATTTAGTAAGGTTCAGGTTAAAACAGCCACAACGGTTCAAACCAACACAGGAGATGCCAAGCAGGTTAGGCTAGGTGGATGCGGTAGGTCCGAATATAAGGATGGAGACTTTGACATTCTTGCGGTTGTGGTTGACGAAGATGTGCTTATTTTCACATGGGACGAAGTAAAAGGTAAGACATCCATGTGTGTCGGCAAGAGAAACAAAGGCATAAAACTATAGGAGAAATTATTATGGCTATGACAAAGAAATTTAAAGTGTCCTTCGACGTTACCGCAAAGATGTCG  >  NC_001604.1/16304‑16904`

  

Alignment Legend

Aligned base mismatch/match (shaded by quality score): `ATCG/ATCG` `< 3 ≤` `ATCG/ATCG` `< 11 ≤` `ATCG/ATCG` `< 24 ≤` `ATCG/ATCG` `< 36 ≤` `ATCG/ATCG` `< 38 ≤` `ATCG/ATCG`

Unaligned base: `atcg`    Masked matching base: `atcg`    Alignment gap: `‑`    Deleted base: `‑`

Reads not counted as support for junction

read\_name Not counted due to insufficient overlap past the breakpoint.

read\_name Not counted due to not crossing MOB target site duplication.
